# Computer-Controlled Virtual Humans in Patient-Facing Systems: Systematic Review and Meta-Analysis

**DOI:** 10.2196/18839

**Published:** 2020-07-30

**Authors:** Debaleena Chattopadhyay, Tengteng Ma, Hasti Sharifi, Pamela Martyn-Nemeth

**Affiliations:** 1 Department of Computer Science University of Illinois at Chicago Chicago, IL United States; 2 Department of Information and Decision Sciences University of Illinois at Chicago Chicago, IL United States; 3 Department of Biobehavioral Health Science University of Illinois at Chicago Chicago, IL United States

**Keywords:** virtual humans, avatars, patient-facing systems, meta-analysis, conversational agents, chatbot, digital interlocutors

## Abstract

**Background:**

Virtual humans (VH) are computer-generated characters that appear humanlike and simulate face-to-face conversations using verbal and nonverbal cues. Unlike formless conversational agents, like smart speakers or chatbots, VH bring together the capabilities of both a conversational agent and an interactive avatar (computer-represented digital characters). Although their use in patient-facing systems has garnered substantial interest, it is unknown to what extent VH are effective in health applications.

**Objective:**

The purpose of this review was to examine the effectiveness of VH in patient-facing systems. The design and implementation characteristics of these systems were also examined.

**Methods:**

Electronic bibliographic databases were searched for peer-reviewed articles with relevant key terms. Studies were included in the systematic review if they designed or evaluated VH in patient-facing systems. Of the included studies, studies that used a randomized controlled trial to evaluate VH were included in the meta-analysis; they were then summarized using the PICOTS framework (population, intervention, comparison group, outcomes, time frame, setting). Summary effect sizes, using random-effects models, were calculated, and the risk of bias was assessed.

**Results:**

Among the 8,125 unique records identified, 53 articles describing 33 unique systems, were qualitatively, systematically reviewed. Two distinct design categories emerged — simple VH and VH augmented with health sensors and trackers. Of the 53 articles, 16 (26 studies) with 44 primary and 22 secondary outcomes were included in the meta-analysis. Meta-analysis of the 44 primary outcome measures revealed a significant difference between intervention and control conditions, favoring the VH intervention (SMD = .166, 95% CI .039-.292, *P*=.012), but with evidence of some heterogeneity, *I^2^*=49.3%. There were more cross-sectional (*k*=15) than longitudinal studies (*k*=11). The intervention was delivered using a personal computer in most studies (*k*=18), followed by a tablet (*k*=4), mobile kiosk (*k*=2), head-mounted display (*k*=1), and a desktop computer in a community center (*k*=1).

**Conclusions:**

We offer evidence for the efficacy of VH in patient-facing systems. Considering that studies included different population and outcome types, more focused analysis is needed in the future. Future studies also need to identify what features of virtual human interventions contribute toward their effectiveness.

## Introduction

Patient-facing systems are digital technologies that offer health services and engage people in their health and wellbeing [1]. These systems promote and facilitate both patient engagement with the health care system and patient empowerment in self-care [2]. Globally, one in three adults suffers from multiple chronic conditions [3]; self-management of chronic conditions has become increasingly complex, requiring sophisticated knowledge, motivation, and skill by patients and their families [4]. Engaging in self-management reduces hospitalization and improves health outcomes and quality of life [5]. Patient-facing systems can facilitate self-management of chronic conditions and fill the gap of time and distance to meet with health care providers. Data increasingly support the value of these patient-facing systems, in the form of mobile health (mHealth) applications [6-9] or sensors to monitor physiological parameters (eg, blood glucose) and behaviors (eg, activity) [10-14]. The latest technological addition to patient-facing systems is computer-controlled virtual humans (VH).

Unlike formless conversational agents, like smart speakers or chatbots, VH bring together the capabilities of both a conversational agent and an interactive avatar (computer-represented digital characters). While their humanlike physical appearance is computer-generated (ie, animated), VH are not human-controlled [15-20] nor cartoonlike [21,22]. They are controlled algorithmically, based on active or passive user input during an interaction. These algorithms can simulate key properties of human face-to-face conversation — both verbal and nonverbal (eg, gaze, emotions, head movements, and metaphoric gestures). While humanlike appearance, movements, and nonverbal behavior offer VH the added advantage of communicating empathy and building rapport, they can also bring psychological and social concerns that may not arise when interacting with formless conversational agents [23-26].

Attempts to make computer interfaces anthropomorphic are not new [27,28]. The sophistication of current digital interlocutors, however, can largely be attributed to the recent advancements in artificial intelligence and computer graphics. Recent variants of anthropomorphic interfaces include relational agents, which are agents designed to build and maintain long-term relationships with users [29], including voice-based, intelligent virtual assistants (eg, Amazon’s Alexa, Microsoft’s Cortana, or Apple’s Siri) and text-based chatbots that run on instant messaging applications [30]. Intricate taxonomies capturing different aspects of conversational agents can be found elsewhere [26,31-33].

While intelligent virtual assistants and chatbots have gained mainstream popularity, VH applications are still in their infancy. As graphic rendering capacities progress and ubiquitous computing peripherals such as virtual reality (VR) and augmented reality head-mounted displays (HMDs) become inexpensive, VH will be increasingly adopted for everyday use. Indeed, similar to education and training [34,35], health care researchers and practitioners have already begun to explore the use of VH in health-related assessments and interventions. As VH come of age and stakeholders in health care deliberate whether to adopt this new computing technology, it is crucial that we examine how prior applications have fared in affecting health outcomes.

To our knowledge, VH in patient-facing systems have not been surveyed before. Only recently, other types of conversational agents in health have been reviewed [31,36-40]. Provoost et al [36] reported a scoping review of 54 articles (49 studies) on low-tech embodied conversational agents in clinical psychology. Laranjo et al [37] surveyed conversational agents with unconstrained natural language input capabilities in health care and included 17 articles, of which only 2 were randomized controlled trials (RCTs). Within mental health, both chatbots ([39]; 10 studies) and relational agents ([38]; 13 studies) have been reviewed. Furthermore, the current state of chatbots and embodied conversational agents as expert systems was recently surveyed ([31]; 40 articles). Kocaballi et al [40] reported a systematic review of how conversational agents can be personalized. It included 13 studies and found that personalization features were assessed for user satisfaction, not in improving health outcomes. None of these reviews included a meta-analysis nor focused on VH. The purpose of this study was to conduct a systematic review and meta-analysis of VH in patient-facing systems.

## Methods

### Overview

This systematic review of the English-language scholarly literature followed standard guidelines for conducting and reporting systematic reviews, including Preferred Reporting Items for Systematic Reviews and Meta-analyses [41] and guidelines from the Cochrane Diagnostic Test Accuracy Working Group [42,43].

### Search Strategy

**Figure 1:**
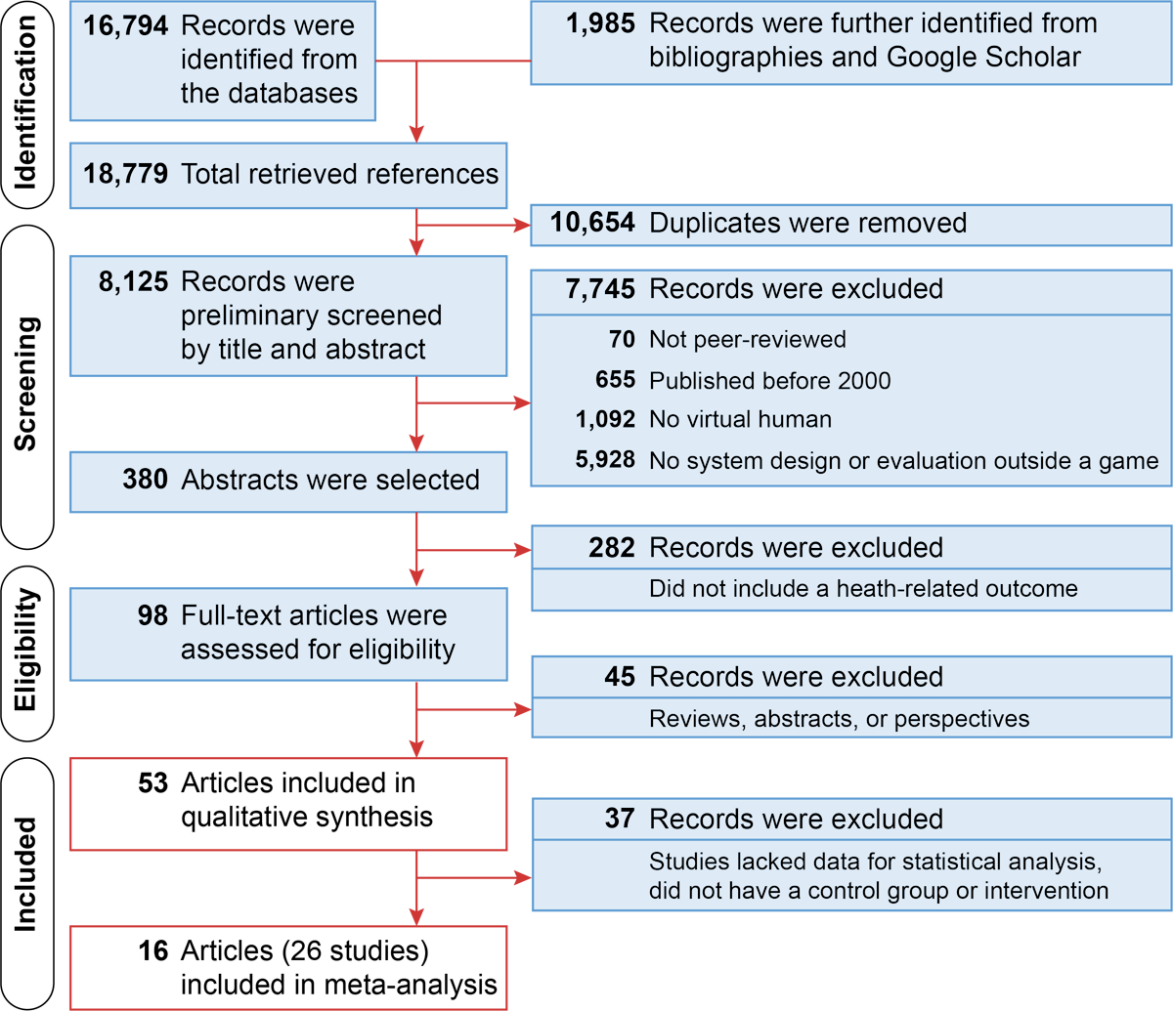
Summary of the literature search.

Literature searches were performed from inception to December 31, 2019 in Google Scholar and 7 online databases: MEDLINE, EMBASE, PsycINFO, CINAHL, Cochrane Central Register of Controlled Trials, PubMed, and ACM Digital Library. Search queries covered 3 domains: (1) avatars, (2) (digital) narratives, and (3) virtual assistants (for details, see Multimedia Appendix 1). Our search was limited to peer-reviewed articles published in English. Manual searches were extended to bibliographies of review articles. Figure 1 shows a summary of the literature search.

### Inclusion and Exclusion Criteria

The topic of our review crosscuts two disciplines — health care and computer science — and their disciplinary priorities are divergent if not orthogonal. On the one hand, health care research prioritizes reporting and replicating empirical evidence of efficacy. On the other hand, driving innovation is a key mission of computer science research. Thus, empirical investigation of these innovative designs — particularly replication of such studies — often takes a back seat. To offer a comprehensive review of the topic at hand, we first present a qualitative, systematic review of VH in patient-facing systems. Some of those articles were then included in a quantitative meta-analysis (Figure 1).

### Qualitative Systematic Review

Studies were included in the qualitative review if they met the following criterion: designed or evaluated VH for health-related outcomes in a patient-facing system. Studies were excluded if they used VH for the training and education of health care professionals or students.

### Meta-Analysis

Some of the articles included in the qualitative synthesis were further included in a meta-analysis if they met the following criteria: (1) compared the effectiveness of VH in a health-related outcome in a target population against a control group with no VH; (2) studied humans of any age; and (3) reported the sample size and mean and variance of the outcome measure in control and experimental groups. Studies were excluded if they did not use a comparator that was equivalent but different from VH (eg, [44]).

### Data Extraction

All records were first downloaded into an EndNote X8.2 library [45], and duplicates were removed. Titles and abstracts were then screened for inclusion and exclusion criteria. To accelerate screening, records were first collated into topics using keyword searches in the EndNote library and then reviewed for inclusion. Two investigators (the first and second authors) independently assessed the relevancy of search results and selected full text articles for further review. The second and third authors independently abstracted the key study factors into a data extraction form and then came to a consensus on which studies met the inclusion criteria; the first author made the definitive decision when discrepancies would arise.

Data from eligible articles were extracted into a spreadsheet. For the qualitative review, data included target population, design objective, type of evaluation, principal findings, and VH characteristics. For the meta-analysis, studies were summarized using the PICOTS (population, intervention, comparison group, outcomes, time frame, setting) framework [46].

### Risk of Bias and Quality Assessment

Articles included in the meta-analysis were assessed for quality and risk of bias using the latest criteria from the Cochrane Consumers and Communication Review Group [47]. Studies were categorized as low risk, unclear, or high risk for each Cochrane bias domain: selection, performance, detection, attrition, reporting, and other [47]. Publication bias was assessed using Egger’s test [48] and a *P*-curve analysis [49] and graphically examined using funnel plots [50].

### Statistical Analysis

All statistical analyses were performed in R using the meta [51] and metafor packages [52,53]. Standardized mean differences (SMD) were used as the effect size to quantify the effectiveness of VH. The overall effectiveness was estimated using a random-effects model, and forest plots were used to graphically present the combined effectiveness. Statistical heterogeneity among the studies was assessed by *I^2^,* which estimates the percentage of total variation across studies due to heterogeneity rather than chance alone. We considered heterogeneity statistically significant at *P*<.05 and used random-effects models to take into account the heterogeneity among the included studies. Subgroup analysis was conducted across studies by the type of health outcome — health and wellbeing as well as attitudes toward health and wellbeing.

## Results

### Qualitative Systematic Review

A total of 16,794 search records were retrieved from the databases, and 1,985 additional records were identified from the bibliographies and Google Scholar. After removing duplicates, we screened the titles and abstracts of 8,125 articles; 380 articles involved a functioning VH system outside a game. Because the computing technology for creating VH did not exist prior to circa 2000, all studies published before 2000 were excluded. These 380 abstracts were further reviewed for their relevance to health, and 282 articles were excluded because the VH was involved in contexts like training, education, or demonstration. The remaining 98 articles underwent full-text review, and 53 articles met the inclusion and exclusion criteria for the qualitative systematic review.

A total of 30 health-related outcomes were identified in the 53 eligible articles, targeting 25 types of populations (Table 1) and 6 modalities of technology delivery apart from a personal computer, desktop, or laptop (Table 2). Unconstrained speech input was sparsely used in VH systems [54,55]. While most systems allowed constrained user input via a touchscreen or keyboard, some systems reacted to nonverbal conversational inputs, such as gaze [56] or proximity [57].

In the 53 eligible articles, 33 unique systems were identified. Of these, 9 systems were used for health assessments and the rest for health interventions (Table 3). Two broad design categories emerged — simple VH and VH with health sensors or trackers (Table 3). These additional trackers did not augment the interaction capabilities of VH, but provided additional data about users (eg, via a heart rhythm monitor, respiration sensor, or eye tracker).

**Table 1:** In the 53 eligible articles, 30 health-related outcomes and 25 target populations were identified.

Health outcome	Target population	Studies
Improve quality of life	Women with overactive bladder (OAB) symptoms	[58]^a^
Self-manage chronic conditions	Individuals with chronic atrial fibrillation (heart condition)	[59-61]
	Individuals with spinal cord injury	[62]
Engage in physical activity	Older adults	[63]^a^ [64,65]
	Individuals with Parkinson’s disease	[66]
	Inactive older adults with low socioeconomic status	[67]^a^
	Healthy adults (no reported health conditions)	[68,69]^a^[70]
	Individuals with schizophrenia	[71]
Improve mood	Individuals with depression	[72]^a^ [73]
Assess auditory verbal hallucinations (AVH)	Individuals with schizophrenia	[74]
Stress management	Women	[75]
	Individuals with chronic pain and depression	[76]^a^ [77]
Healthy eating	Women	[75]
	Healthy adults (no reported health conditions)	[69]^a^
Improve social skills	Children with autism spectrum disorders (ASD)	[44]
	Individuals with schizophrenia	[78]^a^
Assess PTSD^b^ symptoms	US military service members	[54]^a^ [55]
Assess body image disturbance (BID)	Women on diet (nonclinical)	[79]
Anxiety toward death	Older adults	[80]
Find health-related information online	Individuals with low health and computer literacy	[81]^a^ [82]
Explain health documents	Individuals with low health literacy	[83]^a^ [65,84-86]
Attitude toward regular physical activity	Healthy adults (no reported health conditions)	[87]^a^
Attitude toward breastfeeding	Pregnant women in their third semester	[88]^a^ [89,90]
Attitude toward weight loss	Healthy adults (no reported health conditions)	[91]^a^
Retention of medication knowledge	Individuals with type 2 diabetes mellitus	[92]^a^
Attitudes toward prenatal testing for Down syndrome	Nulliparous women	[93]
Improve medication adherence	Individuals with schizophrenia	[71]
Assess emotion recognition	Adults with ASD	[94]
	Individuals with schizophrenia	[95]^a^
	Children with ASD	[96]
Preconception risk assessment	Women	[97]
Assess the effects of social rejection	Individuals with psychotic disorder	[98]
Assess social attention	Children with ASD	[99]
Assist in deep breathing	Healthy adults (no reported health conditions)	[100]
Substance use counseling	Individuals with alcohol use disorder	[56]
	Individuals with opioid use disorder	[101]
Patient trust	Healthy adults (no reported health conditions)	[102]
Assess social anxiety disorder	Women with high social anxiety	[103]
Alleviate social isolation	Older adults	[57]
Understand the distinction between connective and fatty tissue in the breast	Mammography-eligible middle-aged women (40-74 years old)	[104]
Pill count adherence >80%	HIV-positive African American men who have sex with men	[105]

^a^Studies included in the meta-analysis.

^b^PTSD: post-traumatic stress disorder.

**Table 2:** Technology characteristics identified in the eligible studies.

Technology characteristics	Studies
Unconstrained speech input	[54,55]
Computer at a community center or school	[44,67]
Smartphone	[59-61]
Head-mounted display (HMD)	[74,78,79,98,99,103]
Virtual reality (VR) in a PC or HMD	[74,78,79,96,98,99,103]
Mobile kiosk with a computer	[63,65,84-86,88]
Tablet	[63,65,66,70,76,77,80,88]

**Table 3:** Two broad categories of virtual humans emerged from the 53 articles included in the qualitative review.

Type of use	Number of simple virtual humans	Number of virtual humans with health trackers
Intervention	34 [44,57,58,62-64,67-69,71,72,75-78,80-93,97,101,102,104,105]	9 [56,59-61,65,66,70,73,100]
Assessment	7 [54,74,79,94-96,98]	3 [55,99,103]

#### Virtual Humans for Health Interventions

**Figure 2:**
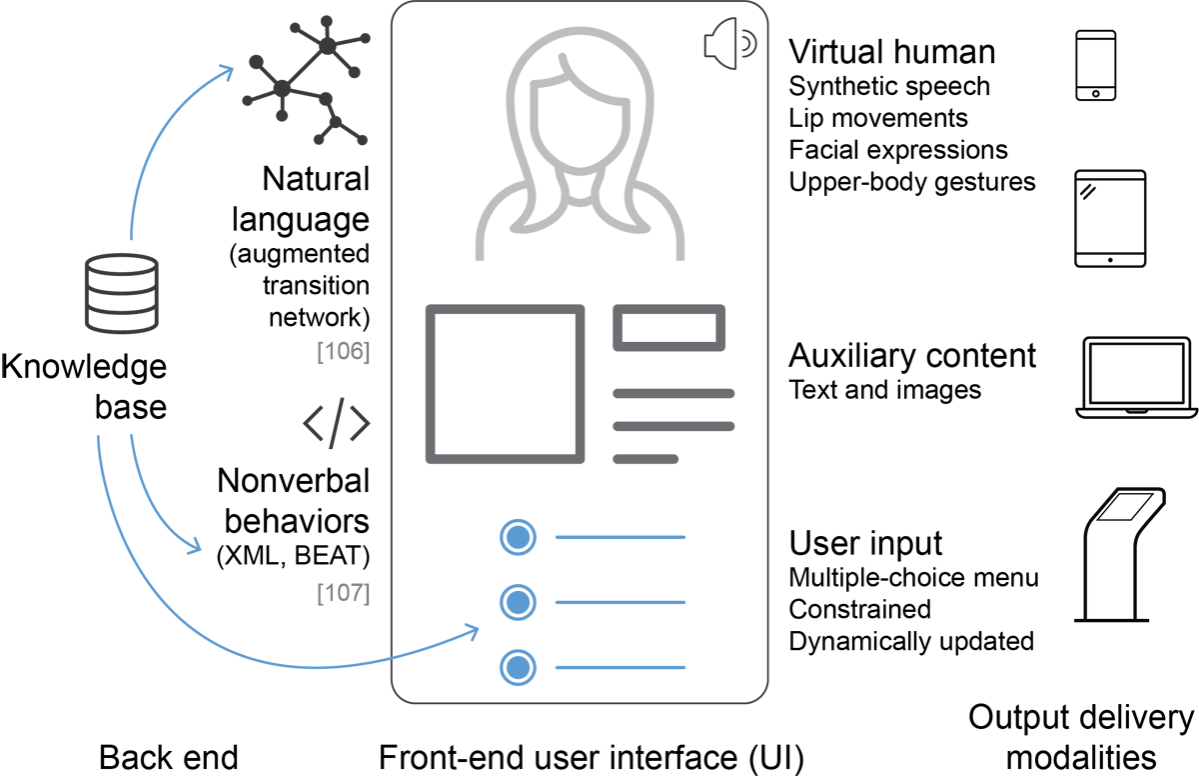
The most common structure of a simple virtual human system designed for health-related interventions. BEAT: Behavior Expression Animation Toolkit.

Of the 34 articles that described simple VH in health-related interventions (Table 3), 27 were based on the same core technology for generating speech [106] and nonverbal behavior [107]. The typical system included a knowledge base of domain-specific top-level dialog fragments (Figure 2). These subdialogs were scripted and then reused to generate natural language speech using a hierarchical transition network, based on augmented transition networks. Augmented transition networks are mathematical structures that can model the grammar of relatively complex natural languages [108]. The generated text was then converted to synthetic speech by the Behavior Expression Animation Toolkit [107]. The Behavior Expression Animation Toolkit also generated appropriate and contextualized nonverbal behavior for the VH. The front end consisted of 3 components: (1) an animated image of the VH communicating with users using speech and gestures, (2) a dynamically updated but constrained multiple-choice menu for user input (via touch or keyboard), and (3) other content supporting the conversation as necessary, like text and images. VH in such a system talked to users with synthetic speech and presented synchronized nonverbal behavior, such as hand gestures and eyebrow raises for emphasis, looking away to signal turn-taking, and posture shifts to mark topic boundaries.

The VH interface in Figure 2 was mostly delivered via smartphones or personal computers. By tailoring the knowledge base, VH were designed for a range of domains, from managing depression [77], through end-of-life planning [80], stress management [75], and educating about breastfeeding [89], to preconception care [97].

Design approaches were varied in the rest of the studies. Dworkin et al [105] followed the same concepts as in Figure 2 but did not discuss implementation details. Two studies used a third-party system [44,72]. Another used VR and an HMD to deliver the VH intervention [78]. Some studies did not detail exactly how the conversations between humans and VH were automated [58,68,78,92], other than mentioning that nonverbal behaviors were programmed to align with verbal utterances. Two studies modeled their intervention after tutorials, with functionality, such as play, pause, repeat, and answering multiple-choice questions [58,92].

Some designs augmented VH with sensor-based tracking (Table 3), such as heart rhythm monitors for managing chronic heart failure [59-61], a pedometer for promoting walking [65,66], and a breathing monitor for assisting in meditation [100].

#### Virtual Humans for Health Assessments

Compared with interventions, fewer studies were found for health-related assessments (Tables 1 and 3); most of these were for people with autism spectrum disorder [94,96,99] and psychotic disorders [74,98,103]. Other studies used VH to assess PTSD symptoms [54,55], emotion recognition [95], and body image disorder [79]. Six studies used VH in a VR environment; 5 used HMDs [74,79,98,99,103], while 1 used a computer [96]. Two studies used advanced tracking systems; these tracking modules were more sophisticated than those used in health interventions. To assess social attention in high-functioning children with autism spectrum disorder, researchers used a sensor to measure head orientation and rotational motion [99]. The MultiSense framework was used for the SimSensei Kiosk [55], which tracks an array of perceptual signals, like smile intensity, gaze direction, and lack of facial expressions, and allows unconstrained speech input [54].

#### Virtual Human Characteristics

Overall, the physical appearances of VH were primarily created using 3-dimensional (3D) character modeling and animation software, such as the Unity3D game engine. They were designed to be racially ambiguous [64] or accordant with target users [86]. But Marcos-Pablos et al [95] generated dynamic expressions for VH (eg, anger, happiness, sadness) by first using a laser scanner to obtain a 3D face model and then using animation software. The speech of VH was either synthetically produced [76] or lip-synced to audio-recorded voice narrations [58,92]. Synthetic speech was generated using commercially available software.

Characteristics, personalities, and mannerisms of VH were manipulated to build rapport with end users [102]. Schulman et al [87] explored the use of social dialog (small talk) and persuasive arguments (about 30 turns) to change attitude toward exercise. VH argued about the advantages of regular exercise and against statements that emphasized disadvantages. Friederichs et al [68] used social dialog at the beginning of an intervention and during transitions between different parts. To persuade users, Andrade et al [58] developed a peer avatar — based on the front-face view of the participant — who delivered a tutorial about self-managing overactive bladder symptoms. Motivational interviewing techniques were used by Friederichs et al [68] to promote physical activity in adults.

Another study explored the use of personal stories available on the internet to personalize the VH’s message and change health behavior [91]. These stories were indexed based on participants's personal state of change during weight loss. To promote breastfeeding, lactation education was framed from a feminist perspective [90]; a VH was designed to motivate women in their third semester to follow breastfeeding recommendations by the US Centers for Disease Control and Prevention. This was realized by introducing a feminist introductory and closing script (eg, “I like to think of myself as a fairly progressive woman, and I hope I do not offend you by some of my opinions” or “A lot of people think that breastfeeding represents a dilemma for feminism.”) [90]. Participants who did not self-identify as feminists were significantly less satisfied with the feminist agent than the controls.

Middle-aged Caucasian and African American VH were designed to achieve racial concordance with users [92], but racial concordance did not significantly affect perceived similarity [86]. Researchers also investigated the effects of attire, background image, and alignment on trust. Study participants found a patient-aligned VH explaining an informed consent document more trustworthy than a medical-aligned or federal-aligned VH. Deictic gestures, such as pointing to a document with a finger or open hand, were designed to aid in document explanation [85]. When explaining a medical document, study participants with low literacy were more satisfied with such a VH than a human. Finally, a common theme across many VH designed for health-related interventions was continuity of care, or longitudinal engagement with end users.

#### Design Process

Of the 53 articles, 23 explicitly described their VH design process [55,58,59,68,71,73,75,78,80,82-87,89-93,95,97,104]. The most common design approaches were collaboration with domain experts, qualitative observation of similar human-human interactions, and adoption of public health and governmental guidelines. Some studies conducted focus groups and one-to-one interviews with target users [59,68,90,92,97,104]. Others informed their design decisions from prior literature or theoretical frameworks [58,59,68,71,90,91,93]. Qualitative observations were often videotaped and coded to generate conversation content [55,71,84]. VH for health-related assessments were always designed in collaboration with health care providers but not end users (Table 3).

#### Theory

Only a few papers explicitly mentioned adopting theoretical frameworks to ground their design of VH [56,60,68,70,91,93,100]. The chronic care model was used to design VH for people with chronic illnesses [109]; the following 3 guidelines were adopted: facilitate communication between patients and providers, make patients aware of the latest care guidelines consistent with scientific evidence, and motivate patients to manage their health [60].

Two frameworks of behavior change were used widely — the transtheoretical model (TTM) of health behavior change [110] and motivational interviewing (MI) counseling style [111]. The transtheoretical model operationalizes intentional behavior change with 3 core constructs — stages of change (precontemplation, contemplation, preparation, action, and maintenance), processes of change, and levels of change — and recommends stage-matched interventions. VH could ask questions to determine these stages and offer matched interventions, such as therapeutic alliance [56,70,91]. MI was developed to elicit people’s readiness to change, explore their attitudes toward change, and transition ambivalence toward reducing resistance to behavior change. The conversation style of VH was sometimes designed based on the MI technique [56,68]. One study designed VH to mirror basic user actions [112], like inhaling and exhaling, to promote behavior change [100]. In another study [93], VH played the role of a decision coach to facilitate shared decision making [113,114]. They informed users of all available options, provided detailed information about those options, and finally assisted in choosing one of them.

#### Design Guidelines

Four papers explicitly offered design guidelines for VH [57,60,64,71]. To design VH that can maintain long-term social relationships with end users (ie, relational agents), Bickmore et al [64] recommended developing a model of user-agent relationship; using relational behavior like social dialog, empathy, and humor; maintaining a memory of past interactions; and providing some variability in agent behavior and overall variability in the dialog structure. When designing VH for mental health interventions that are also relational agents, special design considerations may be needed. For patients with schizophrenia, Bickmore et al [71] recommended prolonging the introduction and conclusion phases of the conversation, using clear and concrete language that focuses on real events to reinforce reality, and not relying solely on nonverbal behaviors like a gaze-away gesture. Specific populations, health outcomes, and device modalities may need additional design considerations. When addressing loneliness in older adults, Ring et al [57] recommended VH engage in social interactions, such as small talk and games, help older adults stay connected with friends and family via contemporary technologies, and alleviate mood disorders or loneliness through different types of talk therapy. When designing for smartphones, Bickmore et al [60] recommended keeping interactions short, allowing interruptions during interactions, and using constrained user input.

### Meta-Analysis

A total of 26 studies (16 articles) published between 2000 and December 31, 2019 were eligible for the meta-analysis, targeting 11 types of populations and including 10 studies with healthy adults [68,69,81,83,87], 3 studies with older adults [63,67], 3 studies with women [58,88], 2 studies with individuals with schizophrenia [78,95], and 3 studies with individuals with depression [72,76].

#### Study Characteristics

The included studies comprised approximately 1400 participants across 13 health and wellbeing objectives. The PICOTS information [46] from the 26 studies is available in Multimedia Appendix 2. Outcomes included 44 primary outcomes and 22 secondary outcomes. Of the 26 studies, 9 focused on attitudes toward health and wellbeing [81,83,87,88,91]. There were more cross-sectional (*k*=15) [54,68,81,83,87,88,91,92,95] than longitudinal studies (*k*=11) [58,63,67,69,72,76,78,88]. Longitudinal studies ranged from 1 month to 6 months (see Multimedia Appendix 2). The VH intervention was delivered using a personal computer in most studies (*k*=18), followed by a tablet (*k*=4), mobile kiosk (*k*=2), HMD (*k*=1), and a desktop computer in a community center (*k*=1).

#### Evidence of Intervention Efficacy

As evident from Table 2 and Multimedia Appendix 2, the 26 studies eligible for meta-analysis varied in terms of outcomes, target population, timing, and intervention design. Hence, a random-effects model was used. A random-effects model does not assume that the estimated effects come from a single homogeneous population, but that true effect sizes vary from study to study. Hedges’ *g* was calculated for each reported outcome, and SMD were used as the effect size to quantify the overall evidence for VH interventions. Next, we report the meta-analysis of the 44 primary outcomes across 26 studies. A meta-analysis of all 66 outcomes (including 22 secondary outcomes) is available in Multimedia Appendix 2.

**Figure 3:**
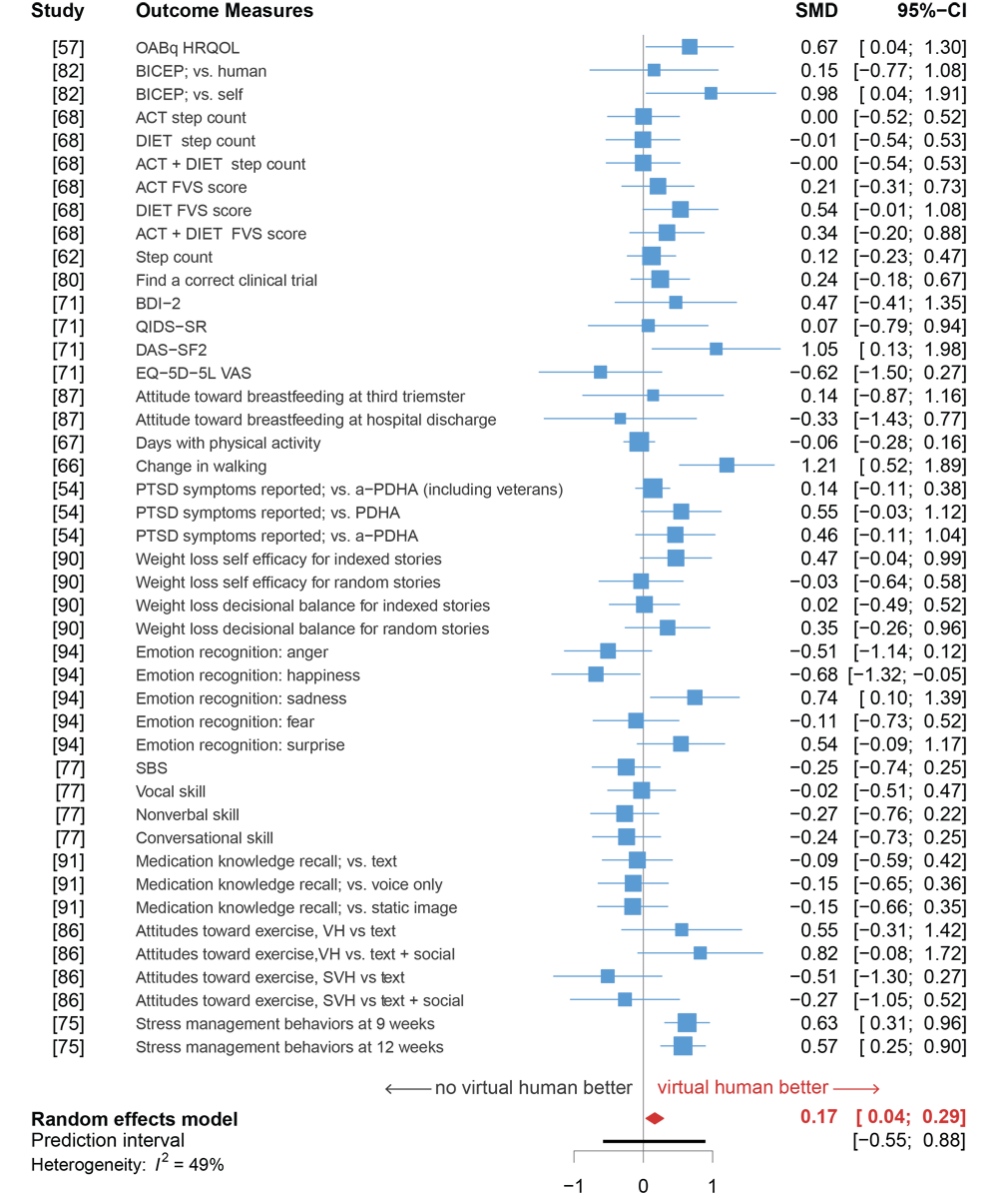
Forest plot of the meta-analysis of health-related virtual human interventions from 26 studies (44 primary outcomes). a-PDHA: anonymized post-deployment health assessment; ACT: physical activity; BDI-2: Beck Depression Inventory-II; BICEP: brief informed consent evaluation protocol; DAS−SF2: Dysfunctional Attitude Scale-Short Form 2; DIET: fruit and vegetable consumption; EQ−5D−5L VAS: 5-level version of the EuroQol 5D visual analogue scale; FVS: NIH/NCI Fruit and Vegetable Scan; HRQOL: health-related quality of life; OABq: overactive bladder questionnaire; PDHA: post-deployment health assessment; PTSD: post-traumatic stress disorder; QIDS−SR: Quality of Life Enjoyment and Satisfaction Questionnaire-Short Form; SBS: social behavior scales; SMD: standardized mean difference; SVH: social virtual human.

The meta-analysis of data from 26 studies (44 outcomes) revealed a significant difference between intervention and control conditions, favoring the VH intervention (SMD .166, 95% CI .039-.292, 95% prediction interval –.548 to .879, *P*=.012) but with evidence of some heterogeneity: *I^2^* = 49.3%, 95% CI 28.1%-64.3% (Figure 3).

A 3-level model (level 2: different outcome measures; level 3: different studies) did not capture a significant amount of variability in the data (*P*>.05). Thus, a 2-level model was used. The between-study heterogeneity of the data was moderate: *τ^2^*=.12, *I^2^*=49.3%. We examined whether this heterogeneity was caused by outliers or influential cases [115,116]. No influential cases were detected, but we spotted 3 outliers [67,76,95]. While 2 of these outlier studies found extremely positive evidence about VH efficacy [67,76], 1 study found extremely negative evidence for one of its outcome measures: happiness emotion recognition by patients with schizophrenia [95]. However, the meta-analysis after removing these outliers (*k*=41, SMD .144, 95% CI .028-.260, *P*=.016; *I^2^*=33.7%, 95% CI 2.6%-54.8%) was similar to the original analysis (Figure 3). Because these 3 studies were interesting outliers, but neither error nor influential outliers [117], we chose to retain them.

A subgroup analysis for health-related outcomes and health-related attitudes was conducted, but no significant difference was found in the overall effect between outcome types (*P*=.762). The number of studies was insufficient to conduct subgroup analyses for different population or outcome types.

**Figure 4:**
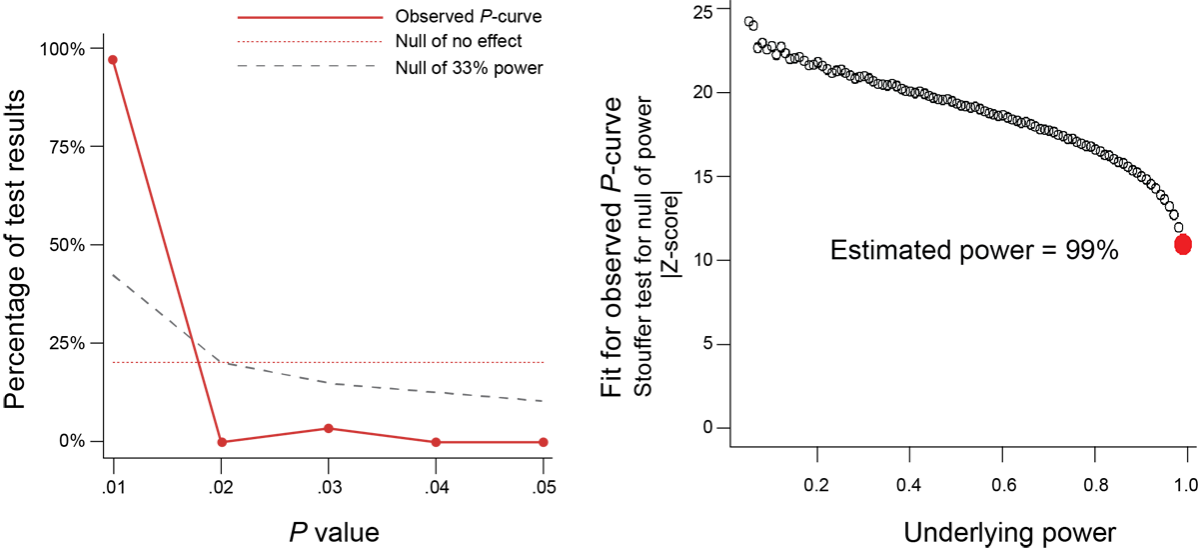
The observed *P*-curve has an estimated power of 99% (left and right), significant right skewness, *P*_full_ < .0001, *P*_half_ < .0001 (left), and no significant flatness, *P*_full_ > .9999, *P*_half_ > .9999.

To explore publication bias, a funnel plot was generated. Egger’s test was not significant (*P*=.70), indicating no substantial asymmetry in the funnel plot. Furthermore, the *P*-curve analysis did not indicate publication bias (Figure 4).

#### Risk of Bias in Included Studies

**Figure 5:**
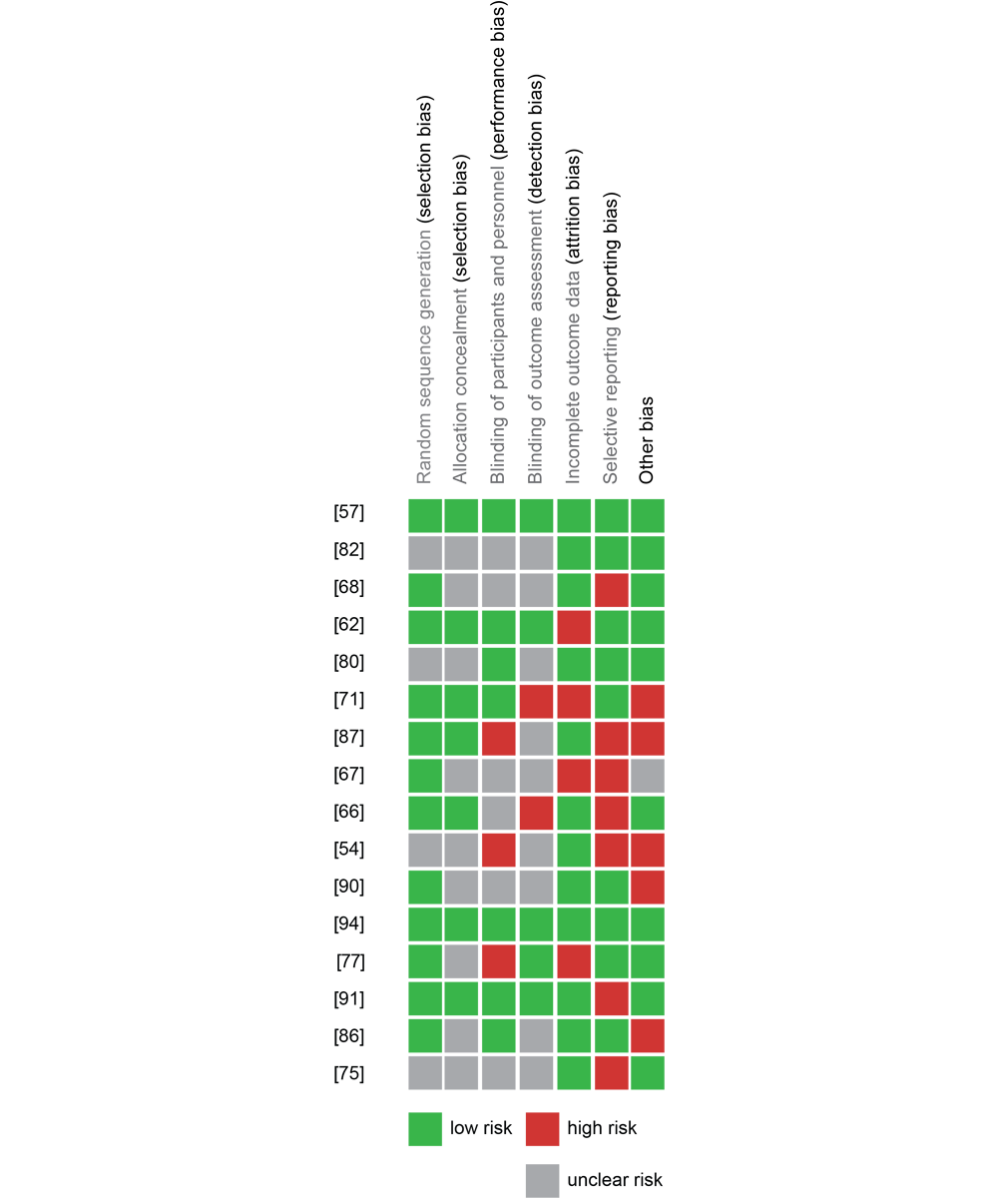
Summary of the authors’ consensus judgment about the risk of bias for each study included in the meta-analysis, by various sources of potential bias.

**Figure 6:**
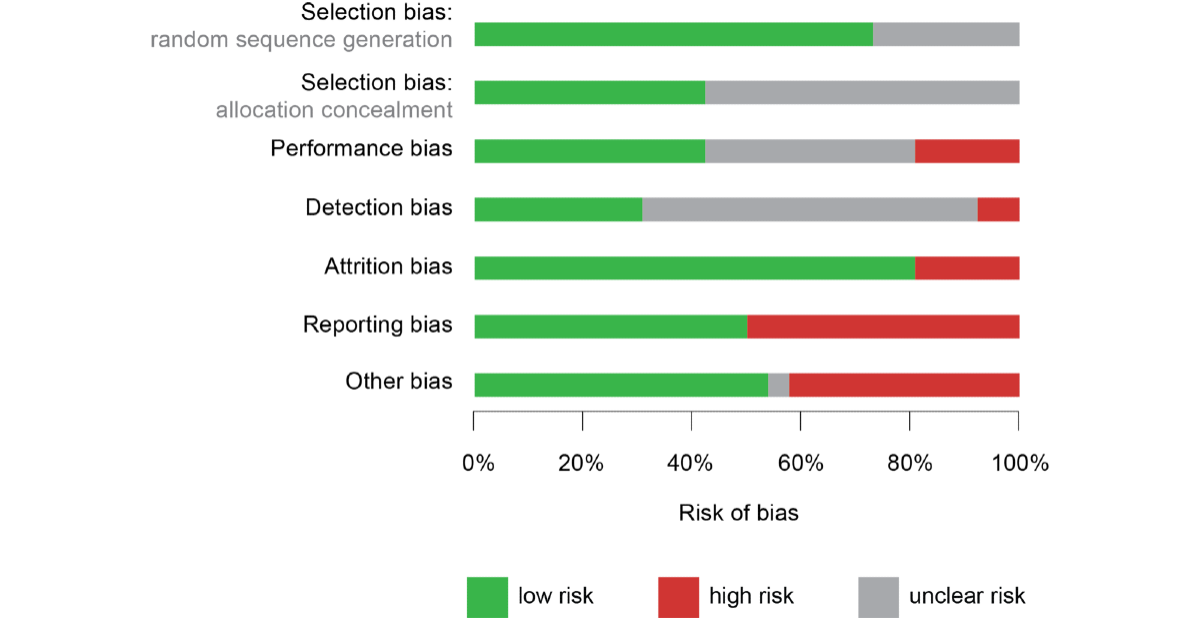
Risk of bias presented as percentages across all 26 studies included in the meta-analysis.

The quality of studies included in the meta-analysis was evaluated for risk of bias (Figures 5 and 6). Randomization was adequate in 19 studies [58,63,67-69,72,78,87,88,91,92,95] but unclear in 7 others [54,76,81,83]. Allocation was reported to be concealed in 11 studies [58,63,67,72,88,92,95], but other studies did not provide enough information to assess allocation bias. It was unclear in 15 studies whether participants and research personnel were blinded to the allocated interventions [54,68,69,76,78,81,83,87,91].

Eleven studies were successful in blinding participants and research personnel to the allocated interventions [58,63,72,81,87,92,95], while 5 studies were not [54,78,88]; the remaining studies were unclear. Blinding the assessors of outcomes was achieved in 8 studies [58,63,78,92,95], 2 studies did not blind research personnel [67,72], and 16 did not clearly report this [54,68,69,76,81,83,87,88,91]. Attrition was high (>20%) in 5 studies [63,68,72,78]. With respect to selective reporting, we identified 13 studies that did not report either descriptive statistics for nonsignificant outcomes or participant demographics [54,67-69,76,88,92]. In assessing other potential sources of bias, we identified 11 studies at a risk of bias due to a small sample size [87,88] or self-selection from provider invitations [72] and websites like Craigslist or Amazon Mechanical Turk [54,69,87,91].

## Discussion

### Primary Findings

This meta-analysis found VH interventions significantly more effective than other types of traditional interventions that did not include conversational agents (SMD .166, 95% CI=.039-.292, *P*=.012). This is an important finding because effective interaction modalities are needed to promote improved consumer or patient engagement in health, thereby promoting behavior change and the management of chronic conditions. The effect size may have been small because the studies involved many different population and outcome types. As more studies become available in the future, some populations and outcome types may show stronger effect sizes than others.

The efficacy of VH may also depend on the type of intervention for which they are used, such as delivering cognitive behavioral therapy vs delivering education. However, the number of current studies was insufficient to conduct such subgroup analyses. Nevertheless, the effectiveness of VH did not significantly differ between health-related outcomes and health-related attitudes.

Like prior reports [37], we found considerable heterogeneity in evaluation methods and outcome measures and a predominance of quasiexperimental study designs or usability studies over RCTs. But unlike previous reviews [36], we found most applications were fully developed, and many of them were either evaluated or soon to be evaluated in RCTs. Our findings cannot be compared directly with the previous reviews because each of the reviews used a different definition of conversational agents or avatars. Typically, earlier reviews did not discriminate between conversational agents with and without a humanlike physical appearance; therefore, they did not discriminate between whether they were able or unable to engage in nonverbal conversation. Only 5 of the 26 studies that were included in the meta-analysis compared the efficacy of VH interventions with text-based chatbots or any other type of conversational agents (Multimedia Appendix 2) [58,68,87,91,92]. Other studies compared interventions against treatment as usual, such as a therapist visit or standard educational materials. Voice-based and text-based conversational agents, without a physical humanlike appearance, were used as comparators in 2 studies [58,92] and 4 studies [68,87,91,92], respectively. Thus, it is too early to tell how much the efficacy of VH in health-related interventions can be attributed to their humanlike physical appearance.

Of course, some health applications, especially some mental health assessments, would only work with VH and could not be replaced with a voice-based or text-based conversational agent. For example, VH were used to assess the influence of unusual voices on daily activities of hallucinating patients [74] and the effect of social rejection in individuals with psychotic disorder [98]. But apart from these, when should a VH intervention be more desirable than other types of conversational agents? Engineering health-related VH interventions is far from trivial and may further cause unintended effects, such as a lack of empathy or a sense of complacency among users [23,24]. When is it worth investing in designing VH over chatbots or smart speakers? This question kept resurfacing in our analysis but remained unanswered. As more studies become available, subgroup analyses of specific interventions could answer this question.

Currently, the prevalence of VH in health applications appears to lag behind that in other areas, such as education and training [118,119] or serious games [120], which is not surprising given the concerns about patient safety [121], ethical and legal issues [122], and perception of professional counselors [123]. Technological advancements will continue to augment VH capabilities; equally important is identifying the design tradeoffs associated with those capabilities in patient-facing systems. When should a conversational agent have a humanlike appearance or any physical embodiment? Would allowing for unconstrained speech and gesture input improve intervention effectiveness or reduce patient safety and privacy? When are nonverbal cues significant in designing a conversational agent? Does age, gender, socioeconomic status, literacy levels, or certain characteristics of target users mediate the effectiveness of VH interventions?

Finally, we found that the input and output of VH systems have evolved significantly over the last 2 decades, drawing on the most recent technological advancements. While systems in the 2000s extensively used desktops, kiosks, 2-dimensional graphics, and constrained text input [64], more recent systems were developed in 3D, were delivered in VR, sometimes used HMDs, and allowed for unconstrained input, such as speech, gestures, and facial emotions. Two key design trends were identified: (1) multimodal sensing of the user’s state using computer vision algorithms and ubiquitous computing technologies, ranging from upper-body gestures to heart rate (input), and (2) striving toward high fidelity, humanlike appearance and behavior of the VH to improve presence and immersion (output).

### Limitations and Future Research

The limitations of this review should be noted and can be addressed by future studies. First, not all studies on VH in patient-facing systems were included in our work. This is because they did not present sufficient quantitative information, only reported usability metrics, or did not clarify whether their avatar technology was computer-controlled or human-controlled. Including additional studies and VH designs could allow reinforcing the results reported here or provide different results. Second, some of the studies included in our meta-analysis had relatively small sample sizes (<20 participants); thus, additional caution is recommended when generalizing these results. Third, there was moderate heterogeneity among trials in the meta-analysis (*τ*^2^=.12, *I^2^*=49.3%.). This can be attributed to the different health outcome measures, population types, and health objectives. For example, health objectives ranged from increasing physical activity and improving mood to improving social skills. When more studies become available with the same or similar health objectives, it would be worth updating this study with new results.

Although research on conversational agents began circa 2000, their design and capabilities have changed and diverged substantially as new technologies and sensors have emerged. This change is expected to continue. Future studies are suggested to consider the difference between different types of conversational agents when synthesizing or generalizing the agents. For example, does a physical appearance or nonverbal behavior increase the effectiveness of a conversational agent? In what kind of tasks? Furthermore, there is rich literature on behavior change and health behavior change theories. However, theoretical frameworks explicating how different features of VH work together in building (or disrupting) rapport with patients is lacking. As such models emerge, future studies will need to examine those relationships between model constructs with methods such as meta-analytic structural equation models.

### Conclusion

VH are conversational agents with a humanlike physical appearance; autonomy in verbal and nonverbal behavior; and speech, gaze, or gesture interaction capabilities. In patient-facing systems, they can demonstrate listening and empathy, as well as tailor to various sociocultural backgrounds, languages, and literacy levels. We surveyed the existing literature on VH in patient-facing systems — from inception to December 2019. Of the 53 articles reviewed, a meta-analysis of 26 studies with more than 1400 participants showed that VH interventions significantly improve health outcomes compared with other traditional intervention methods. But whether a physical embodiment is crucial for a conversational agent to significantly improve health-related outcomes remains to be explored, as does any effect of the VH’s physical appearance, type of voice, or quality of movements.

Although not yet comparable to computer-animated films or high-end video games, the appearance and behavior of VH in health care are increasingly becoming sophisticated, with studies finding that users prefer more humanlike VHs in health care [124,125]. Elsewhere, studies continue to report the possibility of unintended negative user reactions (ie, the uncanny valley effect) when interacting with VH, owing to a mismatch in the levels of realism, either physical, behavioral, or both [23,24,126]. However, the literature on VH in patient-facing systems has not yet examined whether the uncanny valley effect affects patient perception and, in turn, health outcomes.

## Appendix

Multimedia Appendix 1 Literature search details.

Multimedia Appendix 2 PICOTS information.

## Abbreviations

3D: 3-dimensional.
a-PDHA: anonymized post-deployment health assessment.
ACT: physical activity.
ASD: autism spectrum disorders.
ATN: augmented transition network.
BDI-2: Beck Depression Inventory-II.
BEAT: Behavior Expression Animation Toolkit.
BICEP: brief informed consent evaluation protocol.
DAS-SF2: Dysfunctional Attitude Scale-Short Form 2.
DIET: fruit and vegetable consumption.
EQ−5D−5L VAS: 5-level version of the EuroQol 5D visual analogue scale.
FVS: NIH/NCI Fruit and Vegetable Scan.
HMD: head-mounted display.
HRQOL: health-related quality of life.
mHealth: mobile health.
MI: motivational interviewing.
OABq: overactive bladder questionnaire.
PDHA: post-deployment health assessment.
PTSD: post-traumatic stress disorder.
QIDS-SR: Quality of Life Enjoyment and Satisfaction Questionnaire-Short Form.
RCT: randomized controlled trial.
SBS: social behavior scales.
SMD: standardized mean difference.
SVH: social virtual human.
VH: virtual human.
VR: virtual reality.
