# Supplementary material for: Computer-Controlled Virtual Humans in Patient-Facing Systems: Systematic Review and Meta-Analysis
Source: J Med Internet Res. 2020 Jul 30;22(7):e18839. doi: 10.2196/18839 (PMC7426801; doi:10.2196/18839)
Supplement: Multimedia Appendix 1 [file jmir_v22i7e18839_app1.pdf]

Appendix A1

Table A1.1. Keywords used for the literature search.

	Domain	Search terms
#1	Avatars	(avatar or "embodied conversational agent" or "computer-modeled entities" or "digital characters" or "digital interlocutors" or "computer-modeled characters" or "humanlike character" or "anthropomorphic character" or "cartoon character" or "relational agent" or "virtual human")
#2	Narrative	("digital narrative" or "digital health education" or "digital plot" or "health games" or "games for health" or exergames or "healthy games" or "active games" or "active videogame" or "active video game" or "serious games" or "health videogames" or "story immersion")
#3	Virtual assistant	("digital assistant" OR "virtual assistant" OR "digital caregiver" OR "virtual caregiver" OR "virtual nurse" OR "digital nurse")
#4		#1 OR #2 OR #3

Table A1.2. Articles retrieved from the literature search.

Database	Keywords	# of articles retrieved
Cochrane Library	#4	Trials: 768
CINAHL	#4	448
EMBASE	#4	1633
MEDLINE	#4	689
PsycINFO	#4	1626
PubMed	#4	11,563
ACM Digital Library	#4 AND "health"	67
Others		1985
Total		18,779
