# Supplementary material for: Computer-Controlled Virtual Humans in Patient-Facing Systems: Systematic Review and Meta-Analysis
Source: J Med Internet Res. 2020 Jul 30;22(7):e18839. doi: 10.2196/18839 (PMC7426801; doi:10.2196/18839)
Supplement: Multimedia Appendix 2 [file jmir_v22i7e18839_app2.pdf]

Appendix A2

A2.1 PICOTS information from the 26 studies included in the meta-analysis

Table A2.1. PICOTS information from the 26 studies (16 articles) included in the meta-analysis.

Study	Patient population	Intervention	Comparator	Outcome	Timing	Setting
[1]	women (age ≥ 55 years) with symptoms of overactive bladder (OAB) for at least 3 months	self-management program using virtual humans (*n* = 22)	self-management program with voice only (*n* = 19)	[1]self-reported OAB health-related quality of life (OAB HRQOL, [2])<br>[2]patient perception of bladder control (PPBC, [3])<br>[2]geriatric self-efficacy index for urinary incontinence (GSEI-UI, [4])<br>[2]self-reported confidence in doing pelvic floor muscle exercises (PFMEs)<br>[2]self-reported ability to suppress urge<br>[2]urinary frequency/ 24 h<br>[2]urinary urgency / 24 h<br>[2]urge incontinence/ 24 h<br>[2]night-time urination/ day	12-week with interventions at weeks 1, 6, and 12; duration of intervention: 15 – 45 mins	outpatient; independent
[5]	healthy adults (age ≥ 18 years); 45% with		consent document explained by a *human*		one-time intervention;	

Study	Patient population	Intervention	Comparator	Outcome	Timing	Setting
	inadequate health literacy		(*n* = 9)	[1]level of comprehension of the consent document based on BICEP [6]	duration of intervention: open-ended	
			consent document read by *self* (*n* = 11)			
			human	[2]likelihood to sign the consent document		
			self			
		consent document explained by a virtual human (*n* = 9)	human	[2]overall satisfaction with the consent process		at home or research laboratory
			self			
			human	[2]perceived pressure to sign the consent document		
			self			
			self	[2]number of questions or clarifications requested		
		virtual human promoting physical *activity* (*n* = 30)				
		virtual human promoting fruit and vegetable *consumption* (*n* = 27)		[1]pedometer steps		
[7]	healthy adults (age ≥ 18 years); all participants were provided with pedometers	virtual human promoting physical *activity* and fruit and vegetable *consumption* (*n* = 27)	no virtual human (*n* = 27)		2-month intervention daily; duration of intervention: open-ended	at home; independent
		activity		[1]NIH/NCI Fruit and vegetable scan (FVS; [8])		
		consumption				
		activity + consumption				
		activity		[2]BMI		
		consumption				
		activity + consumption				

Study	Patient population	Intervention	Comparator	Outcome	Timing	Setting
[9]	healthy older adults (age ≥ 65); all participants were provided with pedometers	virtual human promoting physical activity (*n* = 100)	no virtual human (*n* = 100)	[1]average daily steps for 30 days before the end of intervention	12 months	at home using personal tablet computers for first 2 months, then using a kiosk computer during outpatient visits
		virtual human promoting physical activity (*n* = 73)	no virtual human (*n* = 55)	[2]average steps per day for 30 days before the end of 2 months		
[10]	adults (age ≥ 18 years); 98% with a current cancer diagnosis	search engine using a virtual human (*n* = 42)	conventional facet- and keyword-based search (*n* = 45)	[1]find a correct clinical trial	one-time intervention lasting about 8 to 12 minutes	at home or research laboratory
[11]	adults with depression	Help4Mood (*n* = 12)	in-person treatment with therapist (*n* = 9)	[1]BDI-2 [12]	four weeks	at home with a laptop computer provided by the researchers
				[1]QIDS-SR [13]		
				[1]DAS-SF2 [14]		
				[1]EQ-5D-5L VAS [15]		
				[1]EQ-5D-5L Utility [15]		
[16]	primipara, pregnant women in the third trimester with one fetus (age ≥ 18)	virtual human (*n* = 7)	no virtual human (*n* = 8)	[1]attitudes toward breastfeeding (IIFAS; [17])	one-time intervention	at prenatal outpatient office visit
		virtual human (*n* = 6)	no virtual human (*n* = 7)		duration of hospital stay post childbirth	at hospital discharge after childbirth

Study	Patient population	Intervention	Comparator	Outcome	Timing	Setting
[18]	healthy adults (age ≥ 18)	motivational interviewing with virtual human (n = 162)	motivational interviewing with text only (n = 146)	[1]number of weekly days with at least 30 minutes of moderate physical activity [19]	one-time intervention; assessment at one-month post intervention	
[20]	older adults (age ≥ 55) not currently engaged in moderate-intensity or moderate activity	virtual human (n = 20)	no virtual human (n = 19)	[1]change in walking behavior, weekly amount of walking over the 4 weeks before the end of intervention in minutes per week	4 months	at a senior center
[21]	US Military service members (active duty)	virtual human interviewer (n = 24)	post-deployment health assessment (PDHA; n = 24)	[1]number of PTSD symptoms reported	one-time intervention	at study site
			anonymized PDHA (n = 24)			
	US Military service members (active duty and veterans)	virtual human interviewer (n = 126)	anonymized PDHA (n = 126)			
[22]	healthy adults (age ≥ 18)	virtual human with indexed stories (n = 35)	text only interface with indexed stories (n = 26)	[1]weight-loss self-efficacy [23]	one-time intervention; duration of intervention: open-ended	online via Amazon's Mechanical Turk
				[1]weight-loss decisional balance [24]		
		virtual human with random stories (n = 19)	text only interface with random stories (n = 23)	[1]weight-loss self-efficacy [23]		
				[1]weight-loss decisional balance [24]		
[25]		virtual humans showing emotions (n = 20)	static images showing emotions	[1]successful emotion recognition—anger	one-time intervention	

Study	Patient population	Intervention	Comparator	Outcome	Timing	Setting
	adults with schizophrenia (age ≥ 18)		(*n* = 20)	[1]successful emotion recognition—happiness<br>[1]successful emotion recognition—sadness<br>[1]successful emotion recognition—fear<br>[1]successful emotion recognition—surprise		at a research laboratory
[26]	adults with schizophrenia (age ≥ 18)	social skills training with virtual human (*n* = 33)	social skills training with no virtual human (*n* = 31)	[1]SBS (overall skill; [27]<br>[1]Vocal skill<br>[1]Nonverbal skill<br>[1]Conversational skill<br>[2]Rathus Assertiveness Schedule [28]<br>[2]Relationship Change Scale [29]<br>[2]Social Problem Solving Inventory-R [30]	10 semiweekly sessions for five weeks	with therapists at an outpatient visit
[31]	male adults with type 2 diabetes mellitus (age ≥ 18)	virtual human based instruction (*n* = 30)	text-based (*n* = 30)<br>voice based (*n* = 30)<br>static image (*n* = 30)	[1]medication knowledge recall	one-time intervention; assessment two weeks after the intervention	at a research laboratory
[32]	healthy adults (age ≥ 18 years)	virtual human promoting physical exercise (*n* = 9)	no virtual human; *text-only* interface (*n* = 13)	[1]self-reported attitude toward exercise	one-time intervention; duration of	at a research laboratory

Study	Patient population	Intervention	Comparator	Outcome	Timing	Setting
			no virtual human; *text-only* interface with *social* dialog (*n* = 12)		intervention: open-ended	
		virtual human promoting physical exercise with social dialog (*n* = 13)	text-only			
			text-only + social			
[33]	adults suffering from chronic pain and depression	group medical visits and virtual human (*n* = 75)	group medical visits (*n* = 79)	[1]self-reported stress management behaviors at 9 weeks	9 weeks of group medical visits and 21 weeks of virtual human based intervention	tablet computer provided to the intervention group
				[1]self-reported stress management behaviors at 21 weeks		

[1]primary outcomes, [2]secondary outcomes; only health-oriented outcomes were considered for the meta-analysis

A2.2 Meta-analysis of data from 26 studies (66 outcomes)

A three-level model did not capture a significant amount of variability in the data ($P > .05$). Thus, a two-level model was used to pool the effect sizes of 66 outcomes (44 primary and 22 secondary). The between-study heterogeneity of the data was moderate, $\tau^2 = .15$, $I^2 = 49.2\%$. Subgroup analyses for health-related *outcomes* [1,7,9,11,18,20,21,25,26,31,33] and health-related *attitudes* were conducted [5,10,16,22,32]. A significant difference was found in the overall effect between the outcome types, $P = .030$.

Meta-analysis of data from 26 studies (66 outcomes) revealed a significant difference between intervention and control conditions favoring the virtual human intervention, *SMD* = .134, 95% CI = .023, .245, 95% prediction interval = −.651, .918, $P = .019$, but with evidence of some heterogeneity, $I^2 = 49.2\%$, 95% CI = 32.4, 61.8 (Figure A2.2).

A subgroup analysis of the 9 studies (20 outcomes) on health-related attitudes also revealed a significant difference between intervention and control conditions favoring the virtual human intervention, *SMD* = .360, 95% CI = .103, .616, 95% prediction interval = −.619, 1.338, $P = .008$, but with evidence of some heterogeneity, $I^2 = 36.5\%$, 95% CI = 0, 62.9.

However, a subgroup analysis of the 17 studies (46 outcomes) on health outcomes did not find a significant difference between intervention and control conditions, $P = .269$.

Figure A2.2. Forest plot of the meta-analysis of health-related virtual human interventions from 26 studies: 44 primary (blue) and 22 secondary outcomes (grey).

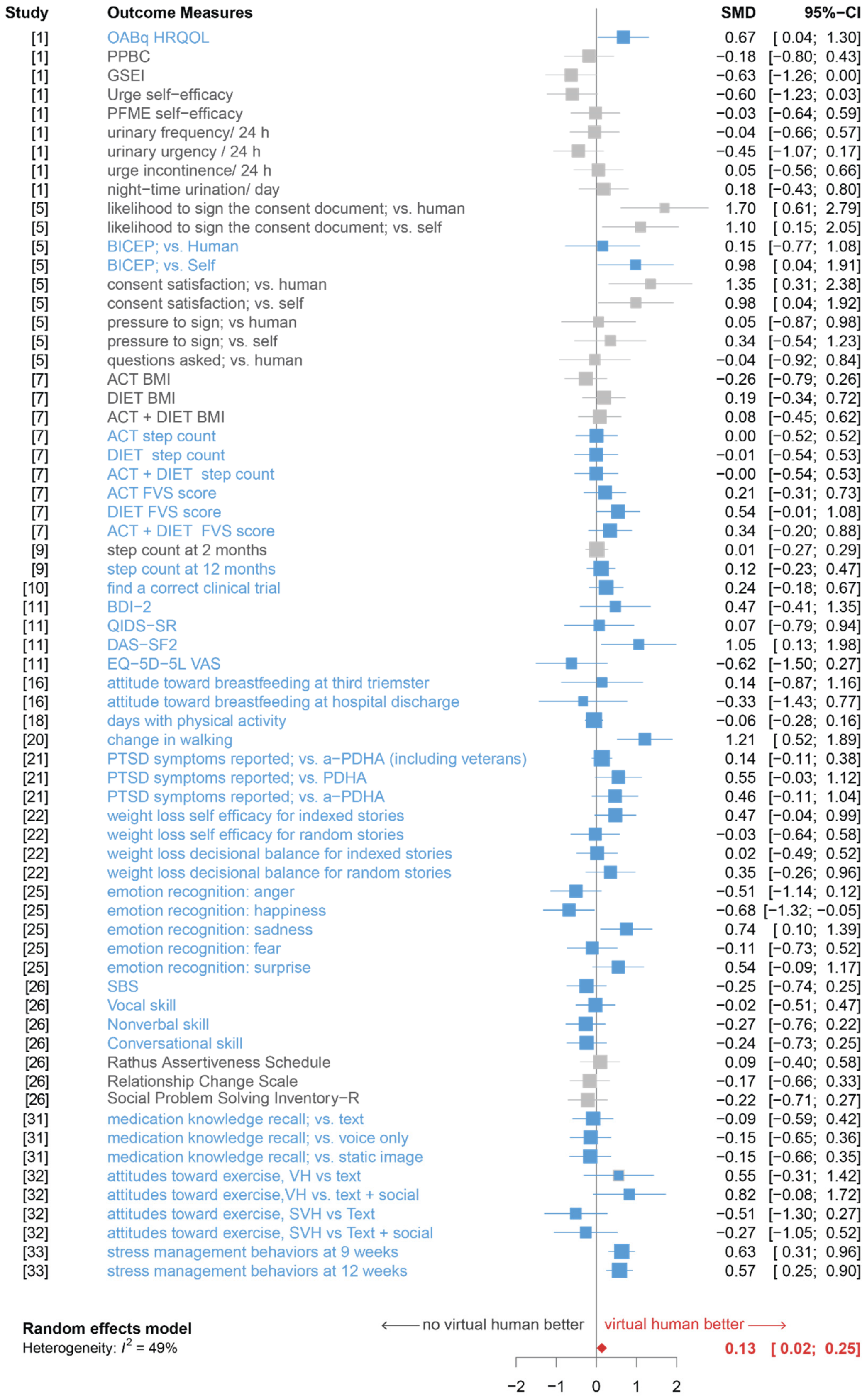
Study
Outcome Measures
SMD
95%−CI
[1] OABq HRQOL 0.67 [0.04; 1.30]
[1] PPBC −0.18 [−0.80; 0.43]
[1] GSEI −0.63 [−1.26; 0.00]
[1] Urge self−efficacy −0.60 [−1.23; 0.03]
[1] PFME self−efficacy −0.03 [−0.64; 0.59]
[1] urinary frequency/ 24 h −0.04 [−0.66; 0.57]
[1] urinary urgency / 24 h −0.45 [−1.07; 0.17]
[1] urge incontinence/ 24 h 0.05 [−0.56; 0.66]
[1] night−time urination/ day 0.18 [−0.43; 0.80]
[5] likelihood to sign the consent document; vs. human 1.70 [0.61; 2.79]
[5] likelihood to sign the consent document; vs. self 1.10 [0.15; 2.05]
[5] BICEP; vs. Human 0.15 [−0.77; 1.08]
[5] BICEP; vs. Self 0.98 [0.04; 1.91]
[5] consent satisfaction; vs. human 1.35 [0.31; 2.38]
[5] consent satisfaction; vs. self 0.98 [0.04; 1.92]
[5] pressure to sign; vs human 0.05 [−0.87; 0.98]
[5] pressure to sign; vs. self 0.34 [−0.54; 1.23]
[5] questions asked; vs. human −0.04 [−0.92; 0.84]
[7] ACT BMI −0.26 [−0.79; 0.26]
[7] DIET BMI 0.19 [−0.34; 0.72]
[7] ACT + DIET BMI 0.08 [−0.45; 0.62]
[7] ACT step count 0.00 [−0.52; 0.52]
[7] DIET step count −0.01 [−0.54; 0.53]
[7] ACT + DIET step count −0.00 [−0.54; 0.53]
[7] ACT FVS score 0.21 [−0.31; 0.73]
[7] DIET FVS score 0.54 [−0.01; 1.08]
[7] ACT + DIET FVS score 0.34 [−0.20; 0.88]
[9] step count at 2 months 0.01 [−0.27; 0.29]
[9] step count at 12 months 0.12 [−0.23; 0.47]
[10] find a correct clinical trial 0.24 [−0.18; 0.67]
[11] BDI−2 0.47 [−0.41; 1.35]
[11] QIDS−SR 0.07 [−0.79; 0.94]
[11] DAS−SF2 1.05 [0.13; 1.98]
[11] EQ−5D−5L VAS −0.62 [−1.50; 0.27]
[16] attitude toward breastfeeding at third triemster 0.14 [−0.87; 1.16]
[16] attitude toward breastfeeding at hospital discharge −0.33 [−1.43; 0.77]
[18] days with physical activity −0.06 [−0.28; 0.16]
[20] change in walking 1.21 [0.52; 1.89]
[21] PTSD symptoms reported; vs. a−PDHA (including veterans) 0.14 [−0.11; 0.38]
[21] PTSD symptoms reported; vs. PDHA 0.55 [−0.03; 1.12]
[21] PTSD symptoms reported; vs. a−PDHA 0.46 [−0.11; 1.04]
[22] weight loss self efficacy for indexed stories 0.47 [−0.04; 0.99]
[22] weight loss self efficacy for random stories −0.03 [−0.64; 0.58]
[22] weight loss decisional balance for indexed stories 0.02 [−0.49; 0.52]
[22] weight loss decisional balance for random stories 0.35 [−0.26; 0.96]
[25] emotion recognition: anger −0.51 [−1.14; 0.12]
[25] emotion recognition: happiness −0.68 [−1.32; −0.05]
[25] emotion recognition: sadness 0.74 [0.10; 1.39]
[25] emotion recognition: fear −0.11 [−0.73; 0.52]
[25] emotion recognition: surprise 0.54 [−0.09; 1.17]
[26] SBS −0.25 [−0.74; 0.25]
[26] Vocal skill −0.02 [−0.51; 0.47]
[26] Nonverbal skill −0.27 [−0.76; 0.22]
[26] Conversational skill −0.24 [−0.73; 0.25]
[26] Rathus Assertiveness Schedule 0.09 [−0.40; 0.58]
[26] Relationship Change Scale −0.17 [−0.66; 0.33]
[26] Social Problem Solving Inventory−R −0.22 [−0.71; 0.27]
[31] medication knowledge recall; vs. text −0.09 [−0.59; 0.42]
[31] medication knowledge recall; vs. voice only −0.15 [−0.65; 0.36]
[31] medication knowledge recall; vs. static image −0.15 [−0.66; 0.35]
[32] attitudes toward exercise, VH vs text 0.55 [−0.31; 1.42]
[32] attitudes toward exercise,VH vs. text + social 0.82 [−0.08; 1.72]
[32] attitudes toward exercise, SVH vs Text −0.51 [−1.30; 0.27]
[32] attitudes toward exercise, SVH vs Text + social −0.27 [−1.05; 0.52]
[33] stress management behaviors at 9 weeks 0.63 [0.31; 0.96]
[33] stress management behaviors at 12 weeks 0.57 [0.25; 0.90]
Random effects model
Heterogeneity: I^2 = 49%
no virtual human better
virtual human better
0.13 [0.02; 0.25]
−2
−1
0
1
2

References

1. Andrade AD, Anam R, Karanam C, Downey P, Ruiz JG. An Overactive Bladder Online Self-management Program With Embedded Avatars: A Randomized Controlled Trial of Efficacy. *Urology.* 2015;85(3):561-567.
2. Coyne K, Revicki D, Hunt T, et al. Psychometric validation of an overactive bladder symptom and health-related quality of life questionnaire: the OAB-q. *Quality of life research.* 2002;11(6):563-574.
3. Coyne KS, Matza LS, Kopp Z, Abrams P. The Validation of the Patient Perception of Bladder Condition (PPBC): A Single-Item Global Measure for Patients with Overactive Bladder. *European Urology.* 2006;49(6):1079-1086.
4. Tannenbaum C, Brouillette J, Korner-Bitensky N, et al. Creation and Testing of the Geriatric Self-Efficacy Index for Urinary Incontinence. *Journal of the American Geriatrics Society.* 2008;56(3):542-547.
5. Bickmore TW, Pfeifer LM, Paasche-Orlow MK. Using computer agents to explain medical documents to patients with low health literacy. *Patient Education and Counseling.* 2009;75(3):315-320.
6. Sugarman J, Lavori PW, Boeger M, et al. Evaluating the quality of informed consent. *Clinical Trials: Journal of the Society for Clinical Trials.* 2005;2(1):34-41.
7. Bickmore TW, Schulman D, Sidner C. Automated interventions for multiple health behaviors using conversational agents. *Patient Education and Counseling.* 2013;92(2):142-148.
8. Thompson F, Subar A, Radimer K, et al. Performance of two new cognitively enhanced fruit and vegetable short assessment forms. Paper presented at: Proceedings of the fourth international conference on dietary assessment methods2000.
9. Bickmore TW, Silliman RA, Nelson K, et al. A Randomized Controlled Trial of an Automated Exercise Coach for Older Adults. *Journal of the American Geriatrics Society.* 2013;61(10):1676-1683.
10. Bickmore TW, Utami D, Matsuyama R, Paasche-Orlow MK. Improving Access to Online Health Information With Conversational Agents: A Randomized Controlled Experiment. *Journal of Medical Internet Research.* 2016;18(1):e1.
11. Burton C, Szentagotai Tatar A, McKinstry B, et al. Pilot randomised controlled trial of Help4Mood, an embodied virtual agent-based system to support treatment of depression. *Journal of Telemedicine and Telecare.* 2016;22(6):348-355.
12. Beck AT, Steer RA, Brown G. Beck Depression Inventory–II. In. *PsycTESTS Dataset*: American Psychological Association (APA); 1996.
13. Rush AJ, Trivedi MH, Ibrahim HM, et al. The 16-Item quick inventory of depressive symptomatology (QIDS), clinician rating (QIDS-C), and self-report (QIDS-SR): a psychometric evaluation in patients with chronic major depression. *Biological Psychiatry.* 2003;54(5):573-583.
14. Beevers CG, Strong DR, Meyer B, Pilkonis PA, Miller IW. Efficiently assessing negative cognition in depression: An item response theory analysis of the Dysfunctional Attitude Scale. *Psychological Assessment.* 2007;19(2):199-209.

15. Herdman M, Gudex C, Lloyd A, et al. Development and preliminary testing of the new five-level version of EQ-5D (EQ-5D-5L). *Quality of Life Research.* 2011;20(10):1727-1736.

16. Edwards RA, Bickmore T, Jenkins L, Foley M, Manjourides J. Use of an Interactive Computer Agent to Support Breastfeeding. *Maternal and Child Health Journal.* 2013;17(10):1961-1968.

17. Mora AdI, Russell DW, Dungy CI, Losch M, Dusdieker L. The Iowa Infant Feeding Attitude Scale: Analysis of Reliability and Validity1. *Journal of Applied Social Psychology.* 1999;29(11):2362-2380.

18. Friederichs S, Bolman C, Oenema A, Guyaux J, Lechner L. Motivational Interviewing in a Web-Based Physical Activity Intervention With an Avatar: Randomized Controlled Trial. *Journal of Medical Internet Research.* 2014;16(2):e48.

19. Wendel-Vos G. Reproducibility and relative validity of the short questionnaire to assess health-enhancing physical activity. *Journal of Clinical Epidemiology.* 2003;56(12):1163-1169.

20. King AC, Bickmore TW, Campero MI, Pruitt LA, Yin JL. Employing Virtual Advisors in Preventive Care for Underserved Communities: Results From the COMPASS Study. *Journal of Health Communication.* 2013;18(12):1449-1464.

21. Lucas GM, Rizzo A, Gratch J, et al. Reporting Mental Health Symptoms: Breaking Down Barriers to Care with Virtual Human Interviewers. *Frontiers in Robotics and AI.* 2017;4.

22. Manuvinakurike R, Velicer WF, Bickmore TW. Automated Indexing of Internet Stories for Health Behavior Change: Weight Loss Attitude Pilot Study. *Journal of Medical Internet Research.* 2014;16(12):e285.

23. Luszczynska A, Scholz U, Schwarzer R. The General Self-Efficacy Scale: Multicultural Validation Studies. *The Journal of Psychology.* 2005;139(5):439-457.

24. O'Connell D, Velicer WF. A Decisional Balance Measure and the Stages of Change Model for Weight Loss. *International Journal of the Addictions.* 1988;23(7):729-750.

25. Marcos-Pablos S, González-Pablos E, Martín-Lorenzo C, Flores LA, Gómez-García-Bermejo J, Zalama E. Virtual Avatar for Emotion Recognition in Patients with Schizophrenia: A Pilot Study. *Frontiers in Human Neuroscience.* 2016;10.

26. Park K-M, Ku J, Choi S-H, et al. A virtual reality application in role-plays of social skills training for schizophrenia: A randomized, controlled trial. *Psychiatry Research.* 2011;189(2):166-172.

27. Trower P, Bryant B, Argyle M. *Social skills and mental health.* University of Pittsburgh Press; 1978.

28. Rathus SA. A 30-item schedule for assessing assertive behavior. *Behavior Therapy.* 1973;4(3):398-406.

29. Schlein S, Guerney B. The relationship change scale (RCS). *Pennsylvania State University.* 1977.

30. D'Zurilla TJ, Nezu AM, Maydeu-Olivares A. Social Problem-Solving Inventory - Revised. In. *PsycTESTS Dataset*: American Psychological Association (APA); 2012.

31. Ruiz JG, Andrade AD, Anam R, Lisigurski M, Karanam C, Sharit J. Computer-Based Programmed Instruction Did Not Improve the Knowledge Retention of Medication

Instructions of Individuals With Type 2 Diabetes Mellitus. *The Diabetes Educator.* 2013;40(1):77-88.

32. Schulman D, Bickmore T. Persuading users through counseling dialogue with a conversational agent. Proceedings of the 4th International Conference on Persuasive Technology - Persuasive '09; 2009.

33. Shamekhi A, Bickmore T, Lestoquoy A, Gardiner P. Augmenting Group Medical Visits with Conversational Agents for Stress Management Behavior Change. In. *Persuasive Technology: Development and Implementation of Personalized Technologies to Change Attitudes and Behaviors*: Springer International Publishing; 2017:55-67.
